# Digital health information-seeking behaviors and trust in AI-based physician chatbots among patients with hypertension: a cross-sectional survey in Saudi Arabia

**DOI:** 10.3389/fpubh.2026.1837642

**Published:** 2026-07-09

**Authors:** Haitham Alzghaibi, Yasir Hayat Mughal, Khurshid Ahmad, Qazi Mohammad Sajid Jamal

**Affiliations:** Department of Health Informatics, College of Applied Medical Sciences, Qassim University, Buraydah, Saudi Arabia

**Keywords:** AI-based physician chatbots, digital health, health information seeking, healthcare, hypertension, information verification

## Abstract

**Background:**

Hypertension requires sustained self-management beyond routine clinical encounters, yet evidence on how patients engage with digital health information and what shapes their trust in AI-based physician chatbots remains limited, particularly in Saudi Arabia.

**Aim:**

This study examined digital health information-seeking behaviors, information verification practices, and perceptions of AI-based physician chatbots among adults diagnosed with hypertension in Saudi Arabia and tested a theoretically grounded model linking six constructs to chatbot safety and trust.

**Methods:**

A cross-sectional online survey was conducted among 322 adults with hypertension recruited through WhatsApp, Telegram, and Facebook patient groups. A structured questionnaire measured six constructs Information Validation Sources (IVS), Digital Self-Diagnosis Tools (DSDT), Online Information-Seeking Behavior (OISB), Familiarity with MOH Digital Services (FMOHDS), Perceived Reliability of Self-Assessment (PRISA), and Chatbot Safety and Trust (CST) adapted from past studies. Internal consistency, descriptive statistics, confirmatory factor analysis, bootstrapping and Kruskal-Wallis group comparisons were performed.

**Results:**

All constructs scored above the scale midpoint (*M* = 3.32–3.78). WhatsApp was the dominant platform for health information seeking (43.5%), verification (51.9%), and communication with health services (41.9%). Measurement model and CFA results shows that scales are reliable and valid, factor loadings, alpha values, AVE and composite reliability and VIF values met threshold. Discriminant validity using Fornell-Larcker is also reported. Bootstrapping results have revealed that OISB has positive significant effect on CST, while PRISA has negative but significant effect on CST, remaining IVS, DSDT and FMOHDS effects on CST were not significant. Moreover, direct effects of predictors on PRISA were also investigated, findings have revealed that FMOHDS is most dominant predictor of PRISA, while OISB negatively but significantly predicted PRISA, however, IVS, DSDT effects on CST were not significant. Regarding indirect mediating effects only PRISA significantly mediates between FMOHDS and CST, while PRISA mediating effects were insignificant between IVS, DSDT OISB and CST.

**Conclusion:**

Trust in AI-based physician chatbots among hypertensive patients is positively shaped by institutional familiarity with MOH digital services and perceived reliability of self-assessment tools and negatively influenced by active independent digital health engagement. The mediating effect of PRISA on FMOHDS and CST suggests that building institutional trust in existing digital infrastructure is a prerequisite for effective AI chatbot adoption. These findings have direct implications for the design of AI-enabled hypertension management strategies within Saudi Arabia’s Vision 2030 digital health agenda.

## Introduction

Hypertension is a highly prevalent and often asymptomatic cardiovascular risk factor, affecting approximately 1.4 billion adults worldwide (1, 2). Even modest reductions in blood pressure confer substantial clinical benefit; for example, a decrease of 10 mmHg systolic and 5 mmHg diastolic blood pressure is associated with roughly 20% lower cardiovascular mortality and marked reductions in stroke, coronary heart disease, and heart failure (3). Despite the availability of effective pharmacotherapy and lifestyle guidelines, global blood pressure control rates remain suboptimal, particularly in low- and middle-income settings where health system capacity and continuity of care are constrained (4, 5). These gaps underscore the need for scalable, patient-centered strategies that can support long-term self-management beyond traditional clinic visits (5, 6).

Through intervention of technologies such as Mobile health (mhealth) emerged as significant methods to enhance hypertension, self-management and supporting adherence to treatment (6–8). It was stated in past studies that intervention through mhealth can reduce systolic BP up to 3-4 mmHg along with improvement in medication, self-care and standard care practices (6, 7, 9). Reductions in systolic BP have been reported in diverse populations, and it has been recommended that mhealth should be integrated into routine care (6, 7). Benefits of mhealth includes better communication between physicians and patients, better health monitoring, and getting accurate BP readings.

Chatbots fall in mhealth ecosystem is a computer-based program, communicate with human beings and which are cost-effective platform offering support to patients with chronic diseases (10–12). Chatbots evaluate the symptoms given to it by human beings. K Health, Ada, and HealthTap are downloaded more than 1 million times from Google (13). Chatbots can facilitate blood pressure self-monitoring, reinforce lifestyle counseling, and enhance hypertension literacy and short-term medication adherence, with ease of use and acceptability (10, 11, 14). Empirical findings from past studies stated that use results in of chatbots 6.5 mmHg reduction in systolic BP is recorded over 8 weeks. (10, 14). However, chatbots effectiveness and acceptance is under evaluation with focus on controlling BP, user engagement and self-management behaviors (11).

Sustained blood pressure (BP) control depends on frequent follow-up visits, tailored feedback, behavioral change counseling, titration support, and frequent clinician-patient interactions (4, 6). Chatbots embedded with physician-like chatbot interfaces within mHealth platforms with continued clinical supervision, are supposed to deliver informed guidelines, treatment plans, context aware recommendations and triaging patients when face-to-face evaluation is required (10). Chatbots can operate in resource-limited settings through messaging ecosystems by maintaining a familiar mode of communication for patients (14, 15). Medication reminders, lifestyle recommendations, and real time health insights are provided using large language models and generative pre-trained transformers. (15, 16). Furthermore, healthcare professionals can get assistance by integrating EHRs and AI-driven clinical decision support in risk stratification, treatment optimization, and formulation of evidence-based treatment plans (16, 17).

In Saudi Arabia, hypertension represents a major contributor to cardiovascular morbidity and mortality and shows substantial variation across regions and age groups (18–20). National survey data indicate an overall hypertension prevalence of about 9.2% among adults aged 15 years and older, with markedly higher rates in older age groups and in regions such as Makkah, underscoring the need for robust, population-level blood pressure control strategies (18, 19). Cardiovascular diseases already account for a large share of deaths in the Kingdom, driven by high rates of obesity, diabetes, dyslipidemia, and hypertension, which together place considerable pressure on the health system and national productivity (19, 21).

Saudi Vision 2030 and the associated Health Sector Transformation Program explicitly prioritize preventive care, non-communicable disease control, and life expectancy gains through digitally enabled, value-based healthcare (22–24). As part of this national agenda, the Ministry of Health has invested heavily in telemedicine, integrated electronic health records, and national mHealth platforms such as Sehhaty and Seha Virtual Hospital, aiming to expand access to specialist services, streamline chronic disease follow-up, and enhance patient engagement across urban and remote regions (22, 23, 25). These initiatives reflect a strategic commitment to transforming healthcare delivery and reducing the disease burden through digital innovation.

Within this national digital transformation, mHealth solutions for hypertension self-management are particularly timely, given the high smartphone penetration and strong patient readiness for app-based blood pressure monitoring and medication support in Saudi hypertensive populations (26–28). Recent Saudi data show that over 85% of surveyed patients with hypertension are willing to use mHealth applications, and most already possess smartphones, suggesting a favorable environment for deploying physician-style chatbots that can deliver personalized counseling, reminders, and triage aligned with Vision 2030’s digital health objectives (26, 27). Embedding physician chatbots into existing national digital platforms could therefore operationalize Vision 2030 goals by strengthening early detection, long-term adherence, and remote follow-up for hypertension, while reducing avoidable clinic visits and improving quality of care at scale (22, 23, 25).

### Study rationale and aims

Although the number of MOH digital platforms has grown rapidly for example, Sehhaty, a consultation service (937) and Chatbot feature(s) in AI, these digital platforms have yet to be studied empirically for how individuals living with hypertension in Saudi Arabia identify, validate, and appraise digital health information, nor how these behaviors inform individuals’ trust of AI-enabled digital health services. As there are substantial gaps in knowledge of these important behaviors, especially in relation to the implementation of AI technologies within the framework of Vision 2030, it is essential to create better solutions for chronic disease management using AI tools based on how, or whether, users will engage effectively. The objective of the current study was to explore the ways that adults diagnosed with hypertension in Saudi Arabia seek, assess, and use digital sources of health information, the forms of sources they rely upon to confirm the validity of this information, and their assessment of the safety of AI-based digital health assistants who act in place of physicians. Additionally, the study will examine predictors of trust and confidence (safety, reliability) in AI-based agents as mediated through the participants’ perceived reliability regarding their own ability to accurately assess their symptoms and health status using online sources and/or self-directed tools. Results will help inform the development of patient-oriented, culturally competent strategies for managing hypertension with AI in line with the digital health agenda set out in Vision 2030. The current study has taken factors like information valid sources (IVS), digital self-diagnosis tools (DSDT), online information seeking behavior (OISB), familiarity with MOH digital services (FMOHDS), perceived reliability of self-assessment, and perceived chatbot safety and trust.

### Hypothesis development and theoretical model

**Figure 1:**
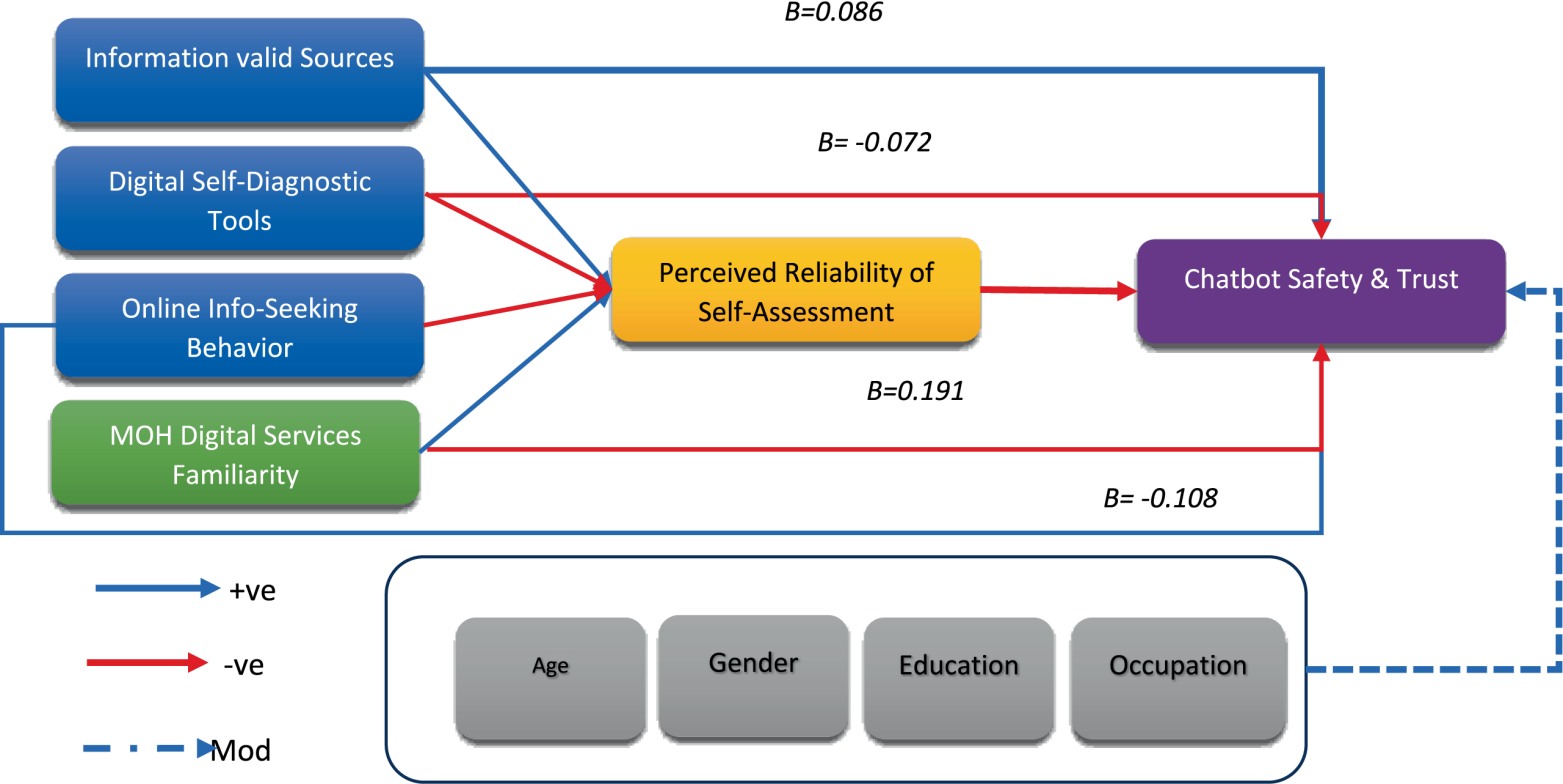
Conceptual framework digital health information seeking behavior and physicians chatbot trust among hypertensive patients in Saudi Arabia.

Health chatbots are getting attention for dealing with risky situations such as health information seeking behavior. Meanwhile it is essential to investigate to what extent the users trust on information taken from chatbots. Use of AI-based physicians’ chatbots theoretical significance lies in Technology Acceptance Model (TAM); Unified theory of acceptance and use of technology (UTAUT). These models have explained how people adopt new technologies and intentions to use and actual use of technology (29). However, Similarity-attraction theory also has explained the use of Chatbots by young people (30). Numerous studies have reported significant effects of various factors affecting chatbot safety and trust (31–33). The factors past studies have used were consumer trust, trust, AI chatbots, Satisfaction, privacy and trust, safety, environment related factors, cognitive trust, social cognition, and immediacy behaviors. Fu et al. (34) conducted systematic literature review and recommended factors such as social media, digital divide, digital self-diagnosis tools, social media influence, online health information seeking behavior, familiarity with digital technologies, psychological factors to be investigated. Studies conducted on information validation sources and digital self-diagnosis tools are significantly related with chatbot safety and trust (35). They also stated benfits of IVS and DISDT, are enhanced acess to health informaiton, early symtom assessment, triage supprot and patient engagement. Study of Elson et al. (36) reported positive impact of familiarity with digital services and chatbot saftey while OHISB also have positive association with CST (37). Form the above dicussion it is obvious that such factors held responsible for CST. These factors are taken as predictors in the current study. Moroever study of Wang et al. (38) expalined in detail perceived reliability of chatbots that ability of chatbot to deliver timely and accurate resposnes. They further elaborated that chatbot’s credibility depends on belief of users’ that chatbot understand their needs and provided the users’ with correct and relevant informaiton. When chatbots provide accurate and relevant informaiton users are mor elikely to perceive chatbots trustworthy. That is why PRISA is taken as mediator to build indirect relaitonship among predictors and criterion variables. See Figure 1. The conceptual framework illustrated in Figure 1 utilizes the Technology Acceptance Model (TAM) proposed by Davis (39), UTAUT2 proposed by Venkatesh et al. (40), and the trust extension model proposed by McKnight et al. (41). The framework illustrates that IVS, DSDT, OISB, and FMOHDS are independent antecedents to the two constructs CST (Chatbot Safety and Trust). A total of five directional hypotheses (H1, H2, H3) hypothesize that IVS, DSDT, and OISB significantly affect CST, while H4 and H5 hypothesize that FMOHDS and PRISA, respectively, significantly affect CST (transfer of institutional trust and perceived reliability will facilitate acceptance of chatbots). Also illustrated in the conceptual framework is a mediation effect in which PRISA mediates between IVS, DSDT, OISB, & FMOHDS and CST Finally, demographics (e.g., age, gender, education, and occupation) are depicted on the edge of the conceptual framework.

*H1:* Information validation sources (IVS) significantly effect on chatbot safety and trust (CST) among patients with hypertension.

*H2:* Digital self-diagnosis tools (DSDT) significantly effect on chatbot safety and trust (CST) among patients with hypertension.

*H3:* Online information-seeking behavior (OISB) significantly effects on chatbot safety and trust (CST) among patients with hypertension.

*H4:* Familiarity with Ministry of Health digital services (FMOHDS) significantly effect on chatbot safety and trust (CST) among patients with hypertension.

*H5:* Perceived reliability of self-assessment (PRISA) significantly effects on chatbot safety and trust (CST) among patients with hypertension.

*H6a:* Information valid sources significantly effect on perceived reliability of self-assessment (PRISA) among patients with hypertension.

*H6b*: Digital self-diagnosis tools significantly effect on perceived reliability of self-assessment (PRISA).

*H6c*: Online information seeking behavior (OISB) significantly effect on perceived reliability of self-assessment (PRISA).

*H6d*: Familiarity with Ministry of Health digital services FMOHDS significantly effect on perceived reliability of self-assessment (PRISA).

*H7a*: Perceived reliability of self-assessment (PRISA) significantly mediates between IVS, and chatbot safety and trust (CST) among patients with hypertension.

*H7b*: Perceived reliability of self-assessment (PRISA) significantly mediates between, DSDT, and chatbot safety and trust (CST) among patients with hypertension.

*H7c*: Perceived reliability of self-assessment (PRISA) significantly mediates between OISB and chatbot safety and trust (CST) among patients with hypertension.

*H7d*: Perceived reliability of self-assessment (PRISA) significantly mediates between FMOHDS and chatbot safety and trust (CST) among patients with hypertension.

*H8:* There are significant mean differences among Demographic characteristics (age, gender, education, and occupation) and digital health engagement constructs and chatbot safety and trust (CST) among patients with hypertension.

## Methods

### Design and setting

This was a quantitative study with a cross-sectional design; a deductive approach was used to examine adults diagnosed with hypertension in Saudi Arabia. This study design is appropriate to assess self-reported behavioral and perceptual data at one specific point in time from a defined population. It is a cost and time-effective approach. Questionnaires were distributed to collect the data using Likert scale. Questionnaires help the researchers to collect the data from large population in short time.

### Participants and sampling

The study’s target population comprises adults aged 18 years or older with a self-reported diagnosis of hypertension who can complete an online questionnaire in either Arabic or English. Participants were excluded if they had one or more chronic conditions to ensure construct homogeneity. Non-probability purposive sampling was used to obtain data from dedicated online communities for hypertensive patients. The reason for using purposive sampling was to select participants with similar traits, i.e., hypertension, to study that group in depth. The population was large, so sampling was crucial. To calculate the sample Krejcie and Morgan (59) table was used. The survey URL was disseminated through Google Forms to five WhatsApp and Telegram groups and one Facebook group for hypertensive patients, as well as a follow-up reminder email every week for two additional weeks to ensure that all respondents completed the survey. The final sample consisted of 322 completed surveys.

### Instruments

Questionnaires were adopted from previous studies whose reliabilities and validities had already been established and reported in the literature. A structured, self-administered questionnaire developed for the assessment of six domains: demographic characteristics, social media platforms use/health messages receipt channels, health information verification/quality criteria, types of health advice sought online, use of digital self-diagnosis tools, & perceptions of safety/trustworthiness with AI-based physician chatbots.

#### Deleted items summary

Based on factor loadings <0.70, one item from OISB, one from DSDT, one from PRISA, and one from CST were removed. Deleted items are listed in Table 1.

**Table 1:** Information of deleted items.

S#	Variable	Item number deleted
1	DSDT	Item no 5 “when I diagnose myself, I seek a diagnosis by requesting a virtual consultation through the “Sehhaty” app’s virtual clinics.
2	OISB	Item no 4 “In general, I regularly search for health-related information online.”
3		Item no 5 “using self-assessment from the Ministry of Health showed that my condition does not require going to the hospital, but I decided to go to the hospital to check on”
4	CST	Item no 3 “the physician chatbot for the Sehaty application is safe so that I can rely on it to diagnose my condition”

#### Measurements

The Six constructs of perception included: Information Validation Sources (IVS), Digital Self-Diagnosis Tools (DSDT), Online Information-Seeking Behavior (OISB), Familiarity with MOH Digital Services (FMOHDS), Perceived Reliability of Self-Assessment (PRISA), & Chatbot Safety & Trust (CST) & were each measured using a five-point Likert scale (1 = strongly disagree to 5 = strongly agree) adapted from (Davis (39), (40), and McKnight et al. (41) trust extension model, modified to fit Saudi Arabia’s digital health context. Multiple response items were utilized where multiple concurrent option selections represent a true reflection of real-world behaviors. Expert review and a pilot user test by 11 patients with hypertension confirmed the content validity of the questionnaire. Reviewers recommended only minor wording changes, without any structural changes. Cronbach’s *α* was used to evaluate internal consistency of all six perceptual constructs, with all six constructs exceeding an *α* ≥ 0.70, or range (0.741–0.899) (see Table 2), resulting in an acceptable level of reliability with one CST item removed.

**Table 2:** Measurement model (confirmatory factor analysis).

Variables	Items	Loadings	*α*	CR (rho_a)	CR(rho_c)	AVE	VIF
IVS	IVS1	0.827	0.899	0.904	0.926	0.714	2.262
	IVS2	0.754					1.716
	IVS3	0.893					3.376
	IVS4	0.864					2.795
	IVS5	0.881					2.825
DSDT	DSDT1	0.870	0.896	0.925	0.925	0.756	3.425
	DSDT2	0.881					3.109
	DSDT3	0.864					2.239
	DSDT4	0.863					2.079
	OISB1	0.835	0.776	0.783	0.871	0.692	1.870
	OISB2	0.774					1.369
	OISB3	0.884					2.034
PRISA	PRISA1	0.722	0.864	0.874	0.908	0.714	1.657
	PRISA2	0.915					3.219
	PRISA3	0.873					2.555
	PRISA4	0.857					2.499
CST	CST 1	0.900	0.741	0.745	0.855	0.666	2.586
	CST 2	0.837					2.305
	CST 4	0.698					1.227
FMOHDS	FMOHDS1	0.933	0.879	0.896	0.914	0.682	4.690
	FMOHDS2	0.760					1.677
	FMOHDS3	0.672					1.812
	FMOHDS	0.841					3.364
	FMOHDS5	0.898					4.481

### Data analysis

Data were analyzed using SPSS (version 22) PLS-SEM (Version 4) & R (version 4.5.2). Descriptive statistics such as frequencies, percentages, means, & standard deviations summarized participant characteristics & construct level responses. A measurement model was developed, and confirmatory factor analysis was run to check the reliability and validity of the scales. In the measurement model, Cronbach’s alpha, composite reliability (CR), average variance extracted (AVE), and variance inflation factor (VIF) were used. Thresholds for these parameters were given by Hair et al., (60); i.e., for Cronbach alpha, it must be >0.70, CR > 0.70, AVE > 0.50, and VIF < 5 (42, 43). Factor loadings must be >0.70 for each item. To test the hypotheses, a structural model was developed. Bootstrapping was run to obtain beta coefficients, standard error, *t*-statistics, and upper and lower limit confidence intervals. Kruskal-Wallis tests were used to examine differences in the composite of constructs among the Groups; Dunn’s *post hoc* comparisons with Bonferroni Correction were applied where statistically significant differences were identified. The effect sizes were reported as eta-squared (*η*^2^). For all analyses, *p* < 0.05 was used as an indicator of statistical significance.

## Result

Table 3 presented the demographic information of the respondents. A total of 322 completed responses were received and used in the statistical analysis of this study. Findings revealed that most of the respondents were male, i.e., 204 (63.4%), and majority of them belong to the age group of 26–35 years, i.e., 140 (43.5%) with graduation, i.e., 232 (72%). Further analysis of results revealed that most of them were government employees, 167 (51.9%), participated in the survey, majority of the respondents use smartphones, i.e., 308 (95.7%), and their preferred language was Arabic, i.e., 253 (78.6%), among them 131 (40.7%) were having advanced IT literacy level and their daily social media usage time is 3-4 h, i.e., 113 (35.1%) Table 3.

**Table 3:** Demographic characteristics of participants (*N* = 322).

Variable	Category	*n*	Percentage
Gender	Male	204	63.4%
	Female	118	36.6%
Age group	26–35 years	140	43.5%
	36–45 years	58	18.0%
	16–25 years	47	14.6%
	46–55 years	39	12.1%
	56–65 years	36	11.2%
	66–75 years	2	0.6%
Education level	Graduate	232	72.0%
	Postgraduate	45	14.0%
	Secondary school	42	13.0%
	Elementary school	2	0.6%
	Primary school	1	0.3%
Occupation	Government employee	167	51.9%
	Unemployed	71	22.0%
	Retired	48	14.9%
	Private sector employee	36	11.2%
Device used	Smartphone	308	95.7%
	Desktop	7	2.2%
	Tablet	5	1.6%
	Laptop	2	0.6%
Preferred language	Arabic	253	78.6%
	English	69	21.4%
IT literacy level	Elementary	143	44.4%
	Advanced	131	40.7%
	Professional	30	9.3%
	Beginner	18	5.6%
Daily social media time	3-4 h	113	35.1%
	4-5 h	95	29.5%
	More than 5 h	64	20.0%
	1-2 h	50	15.5%

Table 4 present digital health and social media use information. Respondents were asked about social media platform used, most of them used WhatsApp, i.e., 140 (43.5%); only 9 respondents use Instagram, and most of them use WhatsApp for verification, i.e., 167 (51.9%); however, only 12 respondents, i.e., 3.7% use Facebook for verifying information. Respondents were asked about outcome of the advice, most of them have selected improved condition with 185 (57.5%); while only 6 (1.9%) have identified some side effects. Google is the first source of search for most of the respondents with *n* = 188 (58.4%); and only 9 respondents use other sources other than physicians, X, calling 937 and YouTube. 52.2% i.e., 168 use WhatsApp as source of health, and majority of them send and receive messages weekly, i.e., 139 (43.2%); WhatsApp is the most used platform for communication by 135 (41.9%) respondents, only 1.6% use email for communication. 113 (35.1%) respondents use the Apps more than once a day.

**Table 4:** Digital health and social media use (*N* = 322).

Variable	Category	*n*	%	Variable	Category	n	%
Social media platform	WhatsApp	140	43.5%	Source of health messages	WhatsApp	168	52.2%
	Facebook	58	18%		Facebook/Instagram	75	23.3%
	YouTube	42	13%		SMS	60	18.6%
	Others	42	13%		Snapchat	19	5.9%
	Snapchat	31	9.6%	Frequency of messages	Weekly	139	43.2%
	Instagram	9	2.8%		Daily	77	23.9%
					Rarely	60	18.6%
					Monthly	46	14.3%
Verification method	WhatsApp	167	51.9%	Communication method	WhatsApp text	135	41.9%
	Instagram	92	28.6%		SMS mobile	120	37.3%
	Twitter/X	28	8.7%		Voice call	38	11.8%
	Google	23	7.1%		Social media (X/Instagram/Snapchat)	24	7.5%
	Facebook	12	3.7%		Email	5	1.6%
Outcome after advice	Improved condition	185	57.5%	App usage frequency	More than once a day	113	35.1%
	No change	64	19.9%		Once a week	65	20.2%
	Did not try medication	59	18.3%		Once a month	48	14.9%
	More side effects	8	2.5%		Never	44	13.7%
	Some side effects	6	1.9%		Just once	21	6.5%
					Once a day	18	5.6%
					Once a year	13	4.0%
First source search	Google	188	58.4%				
	Physician	49	15.2%				
	X	30	9.3%				
	Call 937	24	7.5%				
	YouTube	22	6.8%				
	Other	9	2.8%				

All six constructs scored above (see Table 5) the scale midpoint (3.0), indicating moderate to moderately high endorsement across the sample. Information Validation Sources returned the highest mean (*M* = 3.78, SD = 0.96), followed by Online Information-Seeking Behavior (*M* = 3.71, SD = 0.92), suggesting that participants were most actively engaged in independent online health information practices. Digital Self-Diagnosis Tools (*M* = 3.58, SD = 0.94) and Perceived Reliability of Self-Assessment (*M* = 3.55, SD = 0.82) fell in the mid-range, while Chatbot Safety and Trust (*M* = 3.37, SD = 0.65) and Familiarity with MOH Digital Services (*M* = 3.32, SD = 1.02) recorded the lowest means, indicating comparatively more reserved attitudes toward AI-based physician chatbots and institutional digital service familiarity among this hypertensive population.

**Table 5:** Descriptive statistics for the six study constructs (*N* = 322).

Construct	*N*	Min	Max	Mean	SD
FMOHDS	322	1	5	3.32	1.017
CST	322	1	5	3.37	0.654
PRISA	322	1	5	3.55	0.817
DSDT	322	1	5	3.58	0.942
OISB	322	1	5	3.71	0.925
IVS	322	1	5	3.78	0.955

Among the six constructs measured on a 5-point Likert scale, participants demonstrated the strongest endorsement of online information-seeking behavior, with item means ranging from 3.29 to 3.87 and the highest individual mean recorded for the item assessing general and regular online health information searching (*M* = 3.87, SD = 1.13). Within the same construct, over 66% of respondents agreed or strongly agreed that they search for health-related information online to determine whether a physician visit is warranted (*M* = 3.76, SD = 1.15), suggesting that digital health searching is firmly embedded in participants’ pre-consultation routines. Information validation sources yielded a similarly positive pattern, with participants most inclined to verify online health information through official government-affiliated social media accounts (*M* = 3.82, SD = 1.07) and peer-reviewed scientific research (*M* = 3.68, SD = 1.11), while cross-referencing with friends or family members attracted notably wider disagreement and greater response variability (*M* = 3.25, SD = 1.31). Familiarity with Ministry of Health digital services was moderate to high for established platforms the 937-consultation service (*M* = 3.91, SD = 1.31) and the Sehhaty application (*M* = 3.80, SD = 1.17) yet dropped markedly for the diagnostic self-assessment feature (*M* = 2.90, SD = 1.27) and the automated medical spokesperson service (*M* = 2.71, SD = 1.28), the only two items to fall below the scale midpoint, indicating that awareness does not uniformly extend to newer or less-publicized MOH functionalities (see Table 6).

**Table 6:** Participants’ perceptions of digital health information-seeking, validation practices, ministry of health digital service familiarity, and physician chatbot trust: item-level descriptive statistics (*N* = 322).

Items	Strongly disagree (1)	Disagree (2)	Neutral (3)	Agree (4)	Strongly agree (5)	Mean score	Standard deviation
Information validation sources (IVS)							
When I diagnose myself using the internet, I ensure the reliability of the information by consulting peer-reviewed scientific research.	19 (5.9%)	29 (9%)	64 (19.9%)	132 (41.1%)	77 (24%)	3.68	1.11
When I diagnose myself using the internet, I verify the information by consulting verified health influencers on social media platforms.	12 (3.8%)	27 (8.5%)	94 (29.7%)	111 (35%)	73 (23%)	3.65	1.04
When I diagnose myself using the internet, I confirm the information by referring to official government-affiliated social media accounts.	12 (3.7%)	22 (6.8%)	82 (25.5%)	103 (32%)	103 (32%)	3.82	1.07
When I diagnose myself using the internet, I validate the information by consulting a friend or a family member.	35 (10.9%)	62 (19.3%)	90 (28%)	57 (17.8%)	77 (24%)	3.25	1.31
When I diagnose myself using the internet, I confirm the information by directly consulting a physician.	23 (7.2%)	35 (11%)	87 (27.3%)	103 (32.3%)	71 (22.3%)	3.51	1.16
Digital self-diagnosis tools (DSDT)							
When I diagnose myself, I perform a self-assessment in the “Sehhaty” app to evaluate symptoms related to COVID-19.	35 (10.9%)	30 (9.4%)	93 (29.1%)	77 (24.1%)	85 (26.6%)	3.46	1.28
When I diagnose myself, I perform a self-assessment in the “Sehhaty” app to evaluate other symptoms.	50 (15.5%)	44 (13.7%)	80 (24.8%)	65 (20.2%)	83 (25.8%)	3.27	1.39
When I diagnose myself, I use the medical chatbot in the “Sehhaty” app to assess symptoms.	62 (19.3%)	40 (12.4%)	81 (25.2%)	69 (21.4%)	70 (21.7%)	3.14	1.4
When I diagnose myself, I seek a diagnosis by contacting the 937-consultation service.	37 (11.5%)	27 (8.4%)	83 (25.8%)	87 (27%)	88 (27.3%)	3.5	1.29
When I diagnose myself, I seek a diagnosis by requesting a virtual consultation through the “Sehhaty” app’s virtual clinics.	49 (15.2%)	47 (14.6%)	79 (24.5%)	75 (23.3%)	72 (22.4%)	3.23	1.35
Online information seeking behavior (OISB)							
I resort to searching for health-related information online to determine whether I need to visit a physician.	18 (5.7%)	20 (6.3%)	87 (27.5%)	85 (26.9%)	106 (33.5%)	3.76	1.15
I resort to searching for health-related information online before attending a medical appointment to compare it with the physician’s diagnosis.	43 (13.5%)	38 (11.9%)	99 (31%)	61 (19.1%)	78 (24.5%)	3.29	1.32
I resort to searching for health-related information online after attending a medical appointment to compare it with the physician’s diagnosis.	47 (14.8%)	28 (8.8%)	77 (24.2%)	66 (20.8%)	100 (31.4%)	3.45	1.39
In general, I regularly search for health-related information online.	16 (5%)	21 (6.5%)	71 (22%)	95 (29.5%)	119 (37%)	3.87	1.13
Familiarity with MOH digital services (FMOHDS)							
In general, I am aware of all the technologies provided by the Ministry of Health for diagnosis without going to the hospital	21 (6.5%)	77 (23.9%)	78 (24.2%)	91 (28.3%)	55 (17.1%)	3.25	1.19
I am sufficiently familiar with the Sehaty application, the application of an appointment and their services	0 (0%)	65 (20.2%)	63 (19.6%)	63 (19.6%)	130 (40.5%)	3.8	1.17
I am sufficiently familiar with the 937-consultation service provided by the Ministry of Health	29 (9%)	21 (6.5%)	56 (17.4%)	61 (18.9%)	155 (48.1%)	3.91	1.31
I am familiar enough with the diagnostic self-assessment service in the Sehaty app	52 (16.1%)	65 (20.2%)	125 (38.8%)	23 (7.1%)	57 (17.7%)	2.9	1.27
I am familiar enough with the automated medical spokesperson service for diagnosis in the Sehaty application	68 (21.1%)	70 (21.7%)	120 (37.3%)	15 (4.7%)	49 (15.2%)	2.71	1.28
Perceived reliability and influence of self-assessment (PRISA)							
Using self-assessment from the Ministry of Health with high reliability	6 (1.9%)	44 (13.7%)	146 (45.3%)	62 (19.3%)	64 (19.9%)	3.42	1.01
Using self-assessment from the Ministry of Health made it easier for me to make decisions about my health condition	6 (1.9%)	28 (8.9%)	111 (35.4%)	64 (20.4%)	105 (33.4%)	3.75	1.07
Using self-assessment from the Ministry of Health motivated me to go to the hospital and visit the doctor	0 (0%)	29 (9%)	152 (47.2%)	53 (16.5%)	88 (27.3%)	3.62	0.98
Using self-assessment from the Ministry of Health helped me make the decision not to go to the hospital	0 (0%)	63 (19.6%)	109 (33.9%)	60 (18.6%)	90 (28%)	3.55	1.1
Using self-assessment from the Ministry of Health showed that my condition does not require going to the hospital, but I decided to go to the hospital to check on	7 (2.2%)	77 (23.9%)	135 (41.9%)	42 (13%)	61 (18.9%)	3.23	1.08
Perception on Sehhaty Chatbot, Safety & Trust (CST)							
The Physician chatbot for the Sehaty application is not safe compared to the human doctor	27 (8.4%)	65 (20.2%)	104 (32.3%)	90 (28%)	36 (11.2%)	3.13	1.12
The Physician chatbot in the Sehaty application does not have the power to decide about my health condition compared to the human doctor	6 (1.9%)	40 (12.4%)	117 (36.3%)	55 (17.1%)	104 (32.3%)	3.66	1.11
The Physician chatbot for the Sehaty application is safe so that I can rely on it to diagnose my condition	13 (4%)	38 (11.8%)	184 (57.1%)	59 (18.3%)	28 (8.7%)	3.16	0.89
The Physician chatbot for the Sehaty application is safe, but it is not difficult to rely on it, so I have to talk to the doctor	0 (0%)	12 (3.8%)	163 (51.9%)	97 (30.9%)	42 (13.4%)	3.54	0.77

Responses to the digital self-diagnosis tools and perceived reliability constructs revealed a more cautious engagement profile. Across the five self-diagnosis items, means ranged from 3.14 to 3.46, with the largest proportions of participants selecting the neutral response category, and the Sehhaty chatbot for symptom assessment attracting the highest rate of strong disagreement among all items in this construct (19.3%), reflecting residual hesitancy toward fully automated diagnostic pathways. The perceived reliability items were concentrated around the neutral-to-agree range (*M* = 3.23–3.75), with participants more readily endorsing the view that self-assessment facilitated health decision-making (*M* = 3.75, SD = 1.07) than agreeing that it conclusively justified avoiding hospital attendance (*M* = 3.23, SD = 1.08). Perceptions of the Sehhaty physician chatbot were characterized by conspicuous neutrality: the dominant response across all four items was neutral, accounting for 32.3 to 57.1% of responses, and the lowest standard deviation in the entire instrument was recorded for the item affirming that the chatbot is safe but still necessitates physician consultation (*M* = 3.54, SD = 0.77). Taken together, these distributions suggest that while participants acknowledged the functional utility of AI-based physician chatbots, their trust remains conditional and their comfort with autonomous AI-led diagnosis has yet to reach the threshold required for unqualified acceptance a finding with direct implications for the design and communication strategies surrounding AI-enabled health services.

### Measurement model

A measurement model was developed, and confirmatory factor analysis (CFA) was run in PLS-SEM. Table 2 presents the findings of the CFA. Findings revealed that DSDT’s one item was excluded from the analysis because of low item loadings, i.e., less than 0.70, which creates an issue in overall reliability. Moreover, OISB, PRISA, and CST one item was also excluded due to the same reason; on the other hand, IVS and FMOHDS all five items met the threshold, so no item deletion occurred. From Table 2, it is evident that the Cronbach alpha of all constructs is above 0.70, composite reliability is also higher than 0.70 as per the criteria of (60); average variance extracted is also higher than 0.50, and variance inflation factor (VIF) is less than 5 (60). However, some items and their respective loadings are less than 0.70, but overall reliability and convergent validity are satisfactory, so there is no need to exclude those items from analysis.

Likewise, discriminant validity using Fornell-Larcker criterion (1981) also met the threshold (Table 7) (44). Therefore, it is assumed that the questionnaire used in the study is found reliable and valid.

**Table 7:** Discriminant validity.

Fornell-Larcker	DSDT	FMOHDS	IVS	OISB	CST	PRISA	Perception
DSDT							
FMOHDS	0.166						
IVS	0.885	0.089					
OISB	0.801	0.192	0.753				
CST	0.133	0.210	0.167	0.251			
PRISA	0.181	0.892	0.071	0.244	0.343		
Perception	0.818	0.717	0.779	0.825	0.534	0.753	

Model fit indices are reported in Table 8. RMSEA = 0.089, SRMR = 0.046, TLI = 0.912, CFA = 0.936 met threshold.

**Table 8:** Model fitness CB-SEM.

Model fit indices	Estimated model
Chi-square (*χ*^2^)	396.047
*p*-value	0.000
ChiSqr (*χ*^2^)/d*f*	3.536
RMSEA	0.089
SRMR	0.046
NFI	0.913
TLI	0.912
CFI	0.936

### Structural model

**Figure 2:**
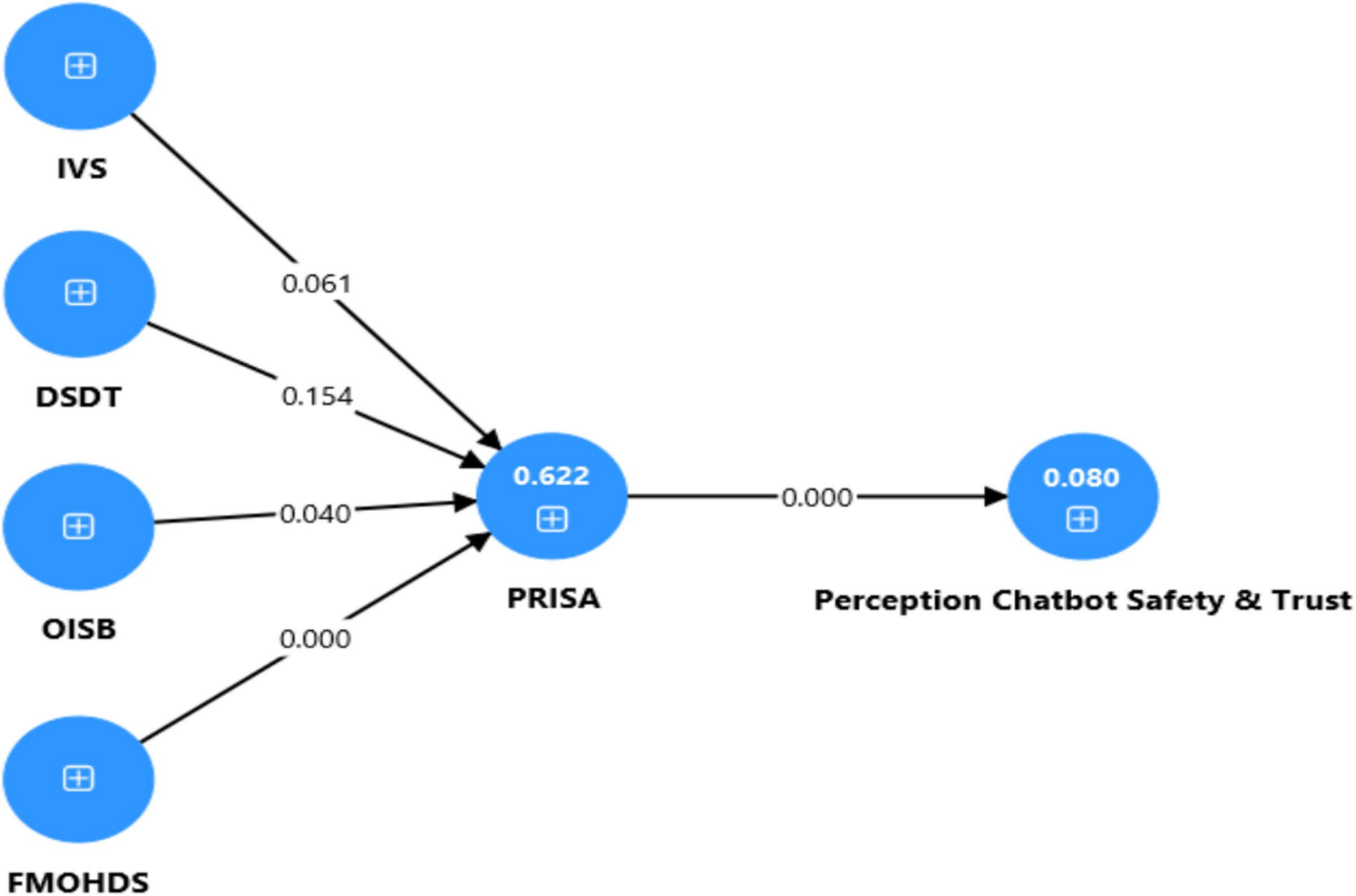
Structural model PLS-SEM.

Structural model was run to test hypotheses. Bootstrapping results are presented in Table 9 and Figure 2. Direct and indirect effects are presented in Table 9 below. Information valid sources (IVS) effect is insignificant on perceived chatbot security and trust (CST) (*β* = 0.086, *p* > 0.05, *t* = 0.924); Thus, H1 is not substantiated and rejected. Likewise, digital self-diagnosis tools (DSDT) also have insignificant impact on CST (*β* = −0.072, *p* > 0.05, *t* = 0.717); this H2 is also rejected. On the contrary, online information seeking behavior (OISB) significantly predicts CST (*β* = 0.191, *p* < 0.05, *t* = 2.523) thus, H3 is substantiated and accepted. Further analysis of results has revealed that familiarity with MOH digital services (FMOHDS) effect is insignificant on CST (*β* = −0.108, *p* > 0.05, *t* = 0.972), thus H4 is rejected. Perceived reliability and influence of self-assessment (PRISA) significantly predict CST (*β* = −0.282, *p* < 0.01, *t* = 4.883), H5 is substantiated and accepted. IVS impact is insignificant on PRISA (*β* = 0.112, *p* > 0.05, *t* = 1.872); H6a is rejected. Similarly, DSDT on PRISA also insignificant (*β* = −0.077, *p* > 0.05, *t* = 1.426), thus, H6b also rejected. OISB and PRISA significant (*β* = −0.096, *p* < 0.05, *t* = 2.056) H6c accepted. FMOHDS (*β* = 0.764, *p* < 0.01, *t* = 32.575) H6d is accepted.

**Table 9:** Direct and indirect effects in PLS-SEM.

Direct effects	*β*	SE	*T*	*p*	LLCI	ULCI	Supprt
IVS → CST	0.086	0.093	0.924	0.356	−0.100	0.264	H1 no
DSDT → CST	−0.072	0.100	0.717	0.473	−0.306	0.103	H2 no
OISB → CST	0.191	0.076	2.523	0.012	0.040	0.329	H3 yes
FMOHDS → CST	−0.108	0.111	0.972	0.331	−0.204	0.381	H4 no
PRISA → CST	−0.282	0.058	4.883	0.000	−0.388	−0.165	H5 yes
IVS → PRISA	0.112	0.060	1.872	0.061	0.024	0.253	H6a no
DSDT → PRISA	−0.077	0.054	1.426	0.154	−0.202	0.009	H6b no
OISB → PRISA	−0.096	0.047	2.056	0.040	−0.191	−0.011	H6c yes
FMOHDS → PRISA	0.764	0.023	32.575	0.000	0.714	0.806	H6d yes
Indirect effects (mediation)							
IVS → PRISA → CST	−0.032	0.018	1.715	0.086	−0.088	−0.007	H7a no
DSDT → PRISA → CST	0.022	0.016	1.333	0.182	−0.002	0.066	H7b no
OISB → PRISA → CST	0.027	0.015	1.801	0.072	0.003	0.064	H7c no
FMOHDS → PRISA → CST	−0.216	0.044	4.947	0.000	−0.295	−0.126	H7d yes

Mediation test was also run to investigate the indirect effects of PRISA on the relationship between predictors (IVS, DSDT, OISB & FMOHDS) and CST. It was found that PRISA did not mediated between IVS and CST (*β* = −0.032, *p* > 0.05, *t* = 1.715); PRISA mediating effect for DSDT and CST (*β* = 0.022, *p* > 0.05, *t* = 1.33) PRISA indirect effect for OISB and CST (*β* = 0.027, *p* > 0.05, *t* = 1.801) thus, H7a, H7b and H7c are rejected, but mediating effect of PRISA between FMOHDS and CST was significant (*β* = −0.216, *p* < 0.01, *t* = 4.947) hence H7d is accepted, The non-significant direct effect (*β* = −0.108, *p* = 0.331) combined with a significant indirect effect (*β* = −0.216, *p* < 0.001) indicates indirect-only mediation, meaning PRISA fully transmits the effect of FMOHDS on CST.”

The findings from the mediation analysis indicate that an individual’s familiarity with Ministry of Health digital services has a strong and positive influence on how reliable they perceive self-assessment to be (*β* = 0.782, *p* < 0.001). (See Table 10. Moreover, PRISA has a strong and negative relationship with one’s trust in and/or sense of safety when using AI-powered physician chatbots (*β* = −0.835, *p* < 0.001). The direct relationship between FMOHDS and CST is also positive and statistically significant (*β* = 0.689, *p* < 0.001) as is the indirect relationship that PRISA has with CST (*β* = −0.653, *p* < 0.001), when direct and indirect effects are significant it means partial mediation exist. PRISA partially mediates the FMOHDS and CST.

**Table 10:** Structural model and mediation analysis results (*R*).

Path/effect	(*B*)	(*β*)	SE	*z*-value	*p*	LLCI	ULCI
FMOHDS → PRISA (*a*)	0.493	0.782	0.057	8.679	<0.001	0.387	0.607
PRISA → CST (*b*)	−1.343	−0.835	0.164	−8.177	<0.001	−1.688	−1.044
FMOHDS → CST (direct, *c*′)	0.699	0.689	0.140	4.987	<0.001	0.470	1.030
Indirect effect (*a* × *b*)	−0.663	−0.653	0.102	−6.514	<0.001	−0.910	−0.493
Total effect (*c*′ + *a* × *b*)	0.036	0.036	0.089	0.411	0.681	−0.130	0.214
Proportion mediated	−18.173	−18.173	641.770	−0.028	0.977	−21249.6	−5.476

*B* = unstandardized regression coefficient; *β* = standardized regression coefficient. SE, Standard error; *p* = significance value; LLCI, lower limit confidence interval; ULCI, upper limit confidence interval.

There were no significant differences between age categories regarding digital health interaction and trust in chatbots in our comparison analysis. Statistically significant comparisons were found between the two occupational categories within the DSDT variable (*p* = 0.026) and the two social media experience categories (*p* = 0.038). (See Table 11). Note that the effect sizes for all these comparisons were quite small. When examining the interactions of demography on the relationships among our study variables (i.e., chatbots and the safety), there was no evidence that there was an interact or moderation effect of gender across the two groups that used chatbots strongly compared weakly (regression lines in Figure 1 are virtually parallel). Neither does this indicate that any of our demographic variables as hypothesized (H8) exert an influence on the relationship among our study variables or that there exists any meaningful variability in our behavioral variables by group.

**Table 11:** Group differences and moderation analysis results.

Grouping variable	Significant construct	Result
Age	None	No significant differences observed
Occupation	DSDT	Significant difference (*p* = 0.026, *η*^2^ = small)
Social media experience	DSDT	Significant difference (*p* = 0.038, *η*^2^ = small)
Gender	None	Interaction not significant; slopes parallel

## Discussion

This study aimed at investigating the mediating effect of PRISA on IVS, DSDT, OISB, FMOHDS, and CST. For this purpose, data was collected from 322 respondents. Moreover, the moderating effect of demographic variables on digital health engagement and perceived chatbot safety and trust is also investigated. For this purpose, structural equation modeling and R software were used for statistical analysis. Findings show that the scales used in the analysis were found to be reliable and valid. However, *post hoc* item deletion occurs due to low factor loadings of a few items, which did not meet the threshold. Additionally, to test direct and indirect hypotheses, bootstrapping was run. Findings indicated that OISB and PRISA have a significant impact on CST, while IVS and FMOHDS have insignificant effects on CST. Regarding mediation, only PRISA mediated between FMOHDS and CST. The findings of this study for IVS insignificantly predicted CST, which means that users do not trust that the chatbot’s information comes from reliable sources. Likewise, the use of digital self-diagnosis tools by users also does not have a meaningful impact on chatbot safety and trust. It means symptoms checker or the use of health apps does not have an impact on the perception of users on how secure the chatbot is or how trustworthy. Similarly, OISB has a significant impact on CST. This means users have shown trust in data protection and privacy regarding chatbot safety and trust. There is a statistically significant relationship between OISB and CST. On the other hand, FMOHDS does not influence CST. Those patients or users who are highly familiar with digital services do not show trust in chatbots. These findings show that chatbot safety and trust are influenced by other factors, such as communication style, user attitudes and behaviors toward AI, and transparency. Simply increasing awareness and familiarity does not bring safety and trust among users. Indirect effects show that PRISA does not mediate between IVS, DSDT, OISB, and CST. But PRISA indirectly mediated between FMOHDS and CST. These findings are in line with the findings of (45–47).

In this research, the digital health information-seeking behavior, information-verification methods, and perspectives of hypertensive patients regarding AI-based physician chatbots were investigated. The findings indicate that the participants in this study represent an engaged population that utilize digital health options via WhatsApp, the primary digital portal for accessing, verifying, and communicating clinically relevant information. The degree of trust in AI-based physician chatbot services is largely dependent on an individual’s familiarity with the healthcare organization/enterprise, coupled with the reliability of the system. Conversely, active, independent engagement with other digital health services is associated with lower trust in AI-based physician chatbots.

WhatsApp is the most popular social media application in Saudi Arabia, with an 89.9% penetration of internet users (48) and 34.5% of internet users use WhatsApp to search for health-related content on PubMed Central. This trend is observed among hypertensive patients in this study, with 43.5% selecting WhatsApp as their primary source of health information, and 51.9% using WhatsApp as their primary method of verifying health information. The co-occurrence of these dual uses has created a circular information ecosystem surrounding health information. Alsaad and AlDossary (49) found that Saudi users often do not determine their message’s credibility prior to sharing it, and educational tools must be developed to educate users regarding misinformation on WhatsApp. As confirmed in our study, approximately 13% of the hypertensive community did not use any verification methods to gather health-related information, and the most sought-after types of health advice were herbal remedies and over-the-counter drugs. Familiarity with the MOH digital service (FMOHDS) is a major predictor but still a significant contributor to PRISA. This finding is supported by a recent comprehensive review of 40 studies on the predictors of trust in the integration of AI and Health Care, which was published in Frontiers of Artificial Intelligence in 2025 and identified the perceived value and perceived utility as the two most important predictors of trust in patients and clinicians alike (50). Therefore, our earlier finding regarding the importance of PRISA in predicting chatbot trust correlates with these findings. When we consider that PRISA mediates the relationship between FMOHDS and chatbot trust, this is also theoretically consistent with the trust extension model proposed by McKnight et al. (41), but has empirical support from a number of the studies we have examined in the context of Saudi Arabia. The qualitative study on the development of the hypertension self-management application in Saudi Arabia identified that the integration of the application into existing MOH infrastructure and culturally adapted content facilitated acceptance of the application by the patient community (51–53), which supports the argument that incorporating AI chatbot features into the Sehhaty platform would enhance the service’s credibility as an extension of an existing health system and, therefore, as a credible source of health information.

On the other hand, OISB PRISA had significant effects on chatbot trust. This finding, which we call the “empowered patient paradox,” is also supported by recent findings from multiple studies. Wu et al. (54) have demonstrated the effects of individualization, distributional structures, and delivery vehicles on healthcare consumers’ perceptions of trustworthiness in providers, and so our finding that FMOHDS is dominant predictor of PRISA and followed by PRISA is a strong predictor of chatbot trust is consistent with those findings (55). Wu et al. (54) conducted a survey of 888 adults with chronic conditions. The data indicates that while previous experiences with chatbots and online health information seeking were highly predictive of using chatbots in the future, participants tended to be doubtful or uncertain about the use of AI chatbots within healthcare. Additionally, most participants did not trust chatbots to provide them with an accurate diagnosis or professional medical advice. PubMed Central. This is somewhat different than the current findings, where there was a negative association between trust and online health information seeking, likely due to the difference in study samples. Participants in Wu et al.’s (54) research were predominantly older, female, and had lower levels of digital engagement in contrast to the current sample, which contained younger individuals who were much more engaged digitally and had higher expectations for performance from AI tools. In Finland, Kauttonen et al. (56) also found that a hesitancy to use chatbots was associated with greater IT skill levels and dislike for interaction with non-humans, even among individuals who are technically adept (57). This further indicates that the level of digital sophistication of an individual does not directly correlate to increased acceptance of chatbots. Research conducted on patients attending primary healthcare clinics in Saudi Arabia showed that online health information greatly influenced patient behavior and communication with physicians and that while respondents indicated positive beliefs about online health resources being accessible and valuable, they also reported that reliable, evidence-based sources remained critically important JMIR. These results are in line with the current study’s high mean IVS (*M* = 3.78), along with the negative impact of IVS on CST. This implies that while patients value validated health-related materials, they do not transfer that valuation to AI-generated clinical materials. An experimental cross-national study across four European countries demonstrated that trust and literacy in AI were the primary predictors of one’s intention to seek AI-based health information through ResearchGate. These findings emphasize that trust in AI health tools is not consistent, but are influenced by the conditions, context, and the user, and reinforce the need for future research on population-specific studies, such as the present study, to guide and shape the implementation of AI physician chatbots.

Credibility and accuracy were rated most frequently as the criteria participants used to evaluate AI health information, by 253 and 195 participants, respectively. The importance placed on Arabic language availability by 121 participants indicates that there is a systematic equity gap in the availability of health-related information, which largely goes unnoticed in Western-dominated frameworks. This highlights the implications for the design of AI chatbot content, as demonstrated by the work of Alhur et al. (58) study on digital health literacy in the Saudi population, where they also found that while digital health practices are increasingly being integrated into daily life, competencies to critically assess the quality of health information available online remain a large competency gap. There is an ongoing need for AI-generated health-related content in Arabic, which meets the standards of credibility and accuracy that the Saudi population considers to be credible.

Only indirect effects of PRISA between FMOHDS and chatbot safety and trust are found significant. All remaining are insignificant. This implies that patients are more confident and able to use digital tools and perceived chatbots are reliable because they are more familiar with ministry of health digital services and have trust on chatbots.

## Conclusion

Based on this study, hypertensive patients’ trust in AI-augmented physicians’ chatbots is largely dependent on their familiarity with institutions and their beliefs regarding the reliability of the system rather than on their digital engagement alone. Patients who are more embedded in the Ministry of Health’s digital health ecosystem are more likely to trust the guidance of a chatbot a finding that has significant implications for the deployment of such chatbots. Embedding the features of physician chatbots into an already trusted platform, such as Sehhaty, could be expected to leverage the existing institutional trust and thereby lower the perceived barrier to the adoption of such technology. On the other hand, the detrimental effects of digital health engagement on chatbot trust indicate the need for a different strategy in engaging these patients; that is, patients who are more informed via current information practices and physician engagement should view AI recommendations as additional to their information acquisition methods rather than a substitute for them. These findings provide original empirical evidence from the GCC region regarding the behavioral and attitudinal precursors to AI physician chatbot trust, thereby filling a significant gap in the literature, which largely encompasses Western patients and healthcare systems, and provides actionable insights to the development of AI-based hypertension management systems that are aligned with the digital health agenda of Saudi Arabia’s Vision 2030.

### Implications

The current study has provided in-depth insights into practice. By considering the complexity and cognitive aspects of using digital health engagement and its constructs, online information seeking behavior, and information from valid sources, this study has provided implications for the quality of health, promotion of public health, empowerment, and trust. First, this study provides an innovative approach for investigating the indirect effects of PRISA on digital health engagement constructs and perception about chatbot safety and trust. This research has built a credible path in which users can build trust in using chatbots. Second, PRISA indirectly enhances the relationship between familiarity with the Ministry of Health digital services and CST.

### Limitations

This study has offered several contributions; however, it is essential to mention the limitations of the study as well. First, this study has used a cross-sectional design, i.e., a single source of data, which can cause bias, and secondly, online recruitment of patients who are disproportionately digitally active. The third limitation is a gender imbalance of 63.4% male. Fourth, the limitation is the sampling technique. Fifth, other factors need to be integrated into the framework used in this study to improve explanatory power and significance. Sixth, the target population is limited, which restricts the generalizability of the findings. The seventh limitation is *post hoc* item deletion, which may influence the underlying structure of the questionnaire and influence the interpretation of the findings. The eight limitation is those participants who were young and have more than one chronic disease been excluded from the study survey, so findings cannot be generalized to those patients having more than one chronic disease, in addition to this, these findings cannot be generalized to older adult(s) less educated, or less digitally engaged hypertensive patients. Likewise, another limitation is self-reported diagnosis of hypertension (no clinical verification). Patients with comorbidities were excluded to maintain construct homogeneity, as the study focused on hypertension-specific digital health behaviors without confounding from other chronic disease management routines

### Future research directions

Future research should add other factors, such as prior experience, ease of use, and social recognition, to simultaneously estimate both direct and indirect effects, use validated scales to measure the extent of trust in chatbots, and employ longitudinal study designs or time-lagged data to examine the process of trust as it develops from exposure to a chatbot platform. Secondly, future studies may use a larger sample size. It is also recommended to validate the model using HLM software.

## Data Availability

The raw data supporting the conclusions of this article will be made available by the authors, without undue reservation.
